# Robotic Resection of an Ectopic Parathyroid Gland: A Systematic Review

**DOI:** 10.7759/cureus.75096

**Published:** 2024-12-04

**Authors:** Shafali Khanom, Katyayani Singh, Lenira S Blinkhorn, Kapilraj Ravendran

**Affiliations:** 1 Medicine, Medical University of Sofia, Sofia, BGR; 2 Medicine, Gradscape, London, GBR; 3 Medicine, MUS (Medical University of Sofia) Surgical Society, Sofia, BGR; 4 General Surgery, East Sussex Healthcare NHS Trust, Brighton and Hove, GBR; 5 General Surgery, Gradscape, London, GBR

**Keywords:** da vinci robotic system, ectopic parathyroid glands, primary hyperparathyroidism, robotic parathyroidectomy, systematic review

## Abstract

Ectopic parathyroid glands result from abnormal migration during development. If not detected promptly, they can lead to persistent or recurrent primary hyperparathyroidism (pHPT). Inferior parathyroid glands are typically located in the anterior mediastinum, while superior parathyroid glands are often near the tracheoesophageal groove, both of which contribute to pHPT. Surgical management of pHPT often involves advanced techniques, with robotic parathyroidectomy using the da Vinci system emerging as an effective approach. This method offers comparable outcomes to traditional surgery, along with enhanced cosmetic results. This systematic review follows PRISMA (Preferred Reporting Items for Systematic Reviews and Meta-analyses) guidelines and an author-approved protocol to compare success rates, outcomes, and complications associated with robotic parathyroidectomy specifically for ectopic parathyroid glands.

The authors conducted a thorough search of PubMed and Google Scholar using Medical Subject Headings (MeSH) terms such as "Robotic resection surgical techniques," "Endocrine surgery," and "Ectopic parathyroid gland." After screening 200 papers, seven studies were selected based on relevance and methodological rigour. Each study's quality and risk of bias were assessed using the Risk of Bias in Non-randomised Studies of Interventions (ROBINS-I) tool for non-randomised studies, and findings were visualised using the Risk of Bias Visualisation (ROBVIS) tool to ensure systematic evaluation of potential biases, such as confounding and selection. This review addresses a gap in the literature by focusing on robotic-assisted surgery for ectopic parathyroid glands, highlighting its advantages, including enhanced visualisation and reduced surgical trauma in challenging anatomical sites. These benefits result in shorter hospital stays, fewer perioperative complications, and improved cosmetic outcomes. However, the broader adoption of robotic surgery requires significant investment in training and equipment, and careful patient selection is essential to minimise complications such as brachial plexus injury. Robotic parathyroidectomy demonstrates favourable patient outcomes in comparison to traditional methods, particularly for difficult-to-access ectopic glands. However, ongoing research is needed to further optimise surgical efficacy, especially through the integration of histopathological and intraoperative monitoring. Future randomised controlled trials (RCTs) should focus on long-term outcomes, cost-effectiveness, and comparing robotic surgery to traditional methods in terms of both clinical success and patient quality of life.

## Introduction and background

Ectopic parathyroid glands result from abnormal migration during embryological development. They are a common cause of persistent or recurrent hyperparathyroidism if not detected early [1]. They can be found in several locations, with inferior parathyroids typically located in the anterior mediastinum and superior parathyroids often near the tracheoesophageal groove [1]. Excessive production of parathyroid hormone (PTH) from enlarged parathyroid glands causes primary hyperparathyroidism (pHPT) [2,3]. pHPT is the third most common endocrine disorder after diabetes and osteoporosis, with a higher prevalence in females [2,3]. In about 25% of pHPT cases, ectopic parathyroid glands are involved, further contributing to the elevated PTH levels [4]. Primary and secondary hyperparathyroidism due to functional ectopic mediastinal glands is more common in patients with persistent or recurrent hyperparathyroidism [4]. It is estimated that approximately 80% of cases result from a single functioning parathyroid adenoma, while the remainder are due to parathyroid hyperplasia or multiple adenomas [5].

It is divided into three types: asymptomatic biochemical type, calculus with urinary tract stones, and bone type with pathological fractures [5]. Operative approaches depend on accurate preoperative localisation of these glands. Transthoracic approaches are often needed for the resection of abnormal parathyroid tissue outside the reach of a cervical incision [6]. Thoracoscopic access is now the preferred method for glands in the thymus or anterior mediastinum [6]. Paraesophageal lesions are displaced by superior parathyroid glands that descend into the chest in the tracheoesophageal groove [6]. Access to this groove is difficult via video-assisted thoracoscopic surgery (VATS) and requires an open approach [7]. A robotic-assisted approach has been used for port-based minimally invasive resection of paraesophageal ectopic parathyroid glands in the superior posterior mediastinum [7]. Thoracoscopic entrance is practical for these patients [8]. The da Vinci robotic system helps with precise dissection in thoracic surgery and offers another option for dissection in the remote mediastinum [8]. Some patients require a median sternotomy or thoracotomy, which can increase morbidity rates [8]. In recent years, robotic parathyroidectomy has become an advanced surgery for pHPT [9]. It offers equivalent results to traditional methods but with better cosmesis [9].

Parathyroidectomy is recommended for pHPT, using various techniques available for minimal and remote access. However, no superior approach has been proven [10]. A new surgical technique called robotic parathyroidectomy, performed with the da Vinci robot, has been found to provide better long-term results than regular endoscopic surgery [10]. The review explores how parathyroid surgery has evolved over time, its benefits and drawbacks, cost-effectiveness, current status, and potential future advancements in the field [10]. In this review, we compare the overall success, outcomes, and any associated complications of robotic resection for an ectopic parathyroid gland [10]. Robotic-assisted parathyroidectomy offers a promising solution for challenging cases involving ectopic parathyroid glands, particularly in difficult-to-reach anatomical locations such as the anterior mediastinum and the tracheoesophageal groove, where traditional approaches are often limited.

While robotic-assisted surgery has been explored for pHPT in general, its specific application for ectopic parathyroid glands remains underrepresented in the literature. This review aims to fill this gap by providing a comprehensive evaluation of robotic-assisted parathyroidectomy for ectopic glands, focusing on success rates, outcomes, and associated complications. This study reviews recent research to assess the benefits and challenges of robotic surgery. It focuses on the unique advantages of robots, like the da Vinci system, which offer clearer views, greater precision, and better cosmetic outcomes than traditional surgery methods.

## Review

Methodology

This study was carried out according to the protocol, which was created and approved individually by all authors and the PRISMA (Preferred Reporting Items for Systematic Reviews and Meta-analyses) guidelines [11]. PRISMA principles were followed in the review [11]. A consensus-building process was used to settle disputes regarding bias and the most appropriate approach to interpret the data, and bias risk was evaluated [11]. A literature search was conducted using the following keywords: "Robotic resection surgical techniques," "Endocrine surgery," and "Ectopic parathyroid gland," utilising Medical Subject Headings (MeSH) Study Selection and Eligible Criteria. The search was conducted using PubMed and Google Scholar. The review protocol was registered at PROSPERO, with registration number CRD42024605841.

Only studies that met the following eligibility criteria were included in this review: case reports, studies that were carefully crafted to guarantee their applicability. We only took into account research on robotic surgery for ectopic parathyroid glands. Studies using non-robotic surgical procedures, articles unrelated to the parathyroid gland, and those with insufficient detail to properly evaluate results were also excluded based on exclusion criteria. We did not include abstracts from conferences, editorials, review articles, studies authored in languages other than English, or studies whose texts were not entirely accessible online.

Data Extraction and Quality Assessment

During the initial screening, all three authors (S.K., K.S., and L.S.) reviewed the titles and abstracts of 200 papers. K.S. and L.S. then conducted the full-text screening, while the first author (S.K.) impartially reviewed the full texts of the selected papers. After applying the inclusion and exclusion criteria, seven relevant papers were identified. The second and third authors (K.S. and L.S.) performed the data extraction, systematically recording key details such as the journal, publication year, study design, outcomes, conclusions, limitations, and implications for each selected paper. The first author (S.K.) reviewed the extracted data to ensure accuracy and consistency, resolving any disagreements between K.S. and L.S. during the screening or bias assessment process. The senior author (K.R.) supervised the overall process, providing guidance and resolving any issues. The first author (S.K.) and the senior author (K.R.) independently assessed the quality of the included studies using the Risk of Bias in Non-randomised Studies of Interventions (ROBINS-I) tool, producing a quality evaluation summary for each publication using the Risk of Bias Visualisation (ROBVIS) tool. Relevant conclusions regarding the benefits and drawbacks of robotic resection procedures for ectopic parathyroid glands were synthesised based on the reviewed articles. The PRISMA guidelines were followed to ensure a methodical and objective evaluation [11].

Results

**Figure 1:**
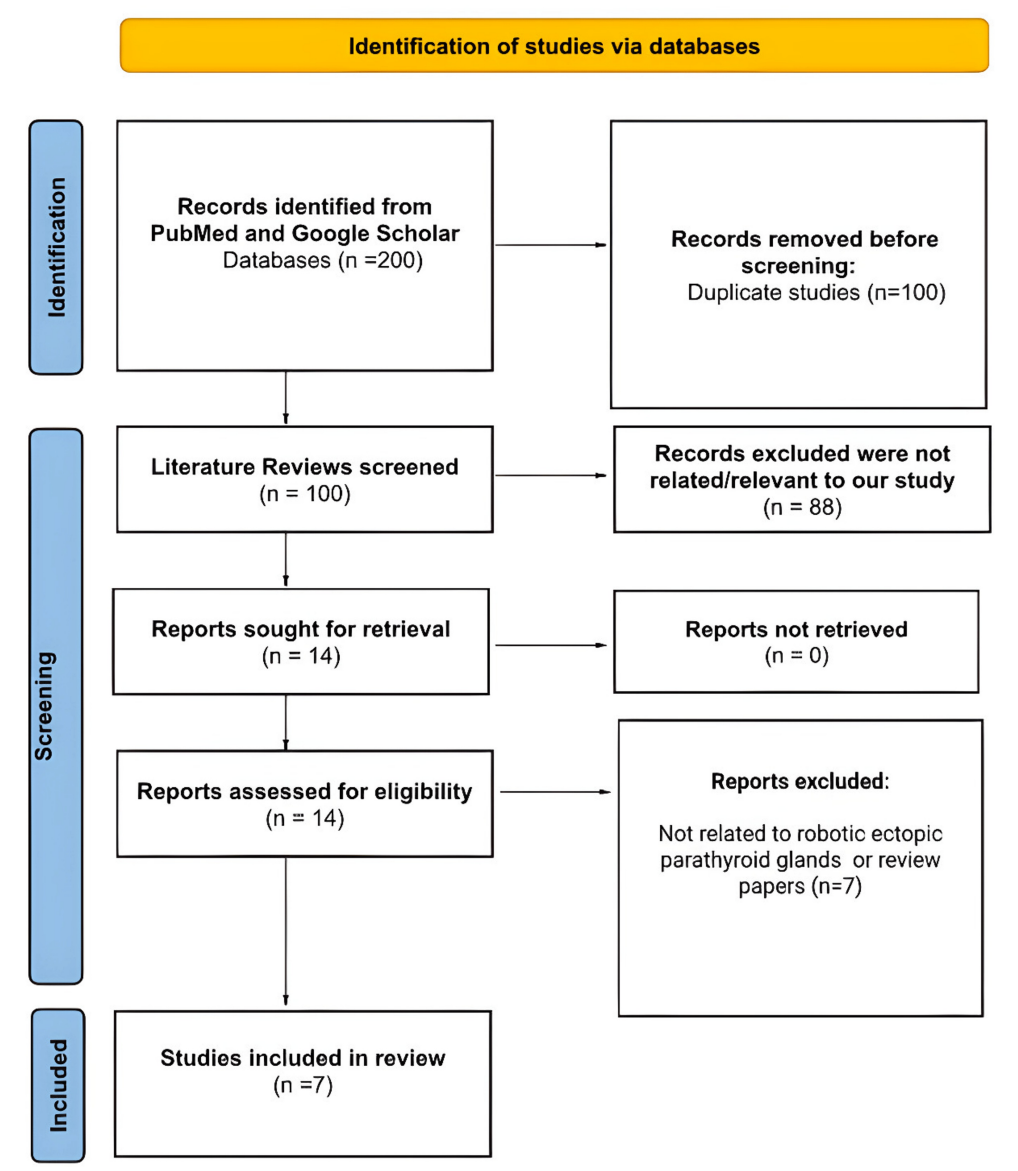
PRISMA flow diagram demonstrating the literature selection strategy. Image credit: [11] PRISMA, Preferred reporting items for systematic reviews and meta-analyses

In the initial database search, 200 items were identified. Of these, 100 were eliminated due to duplication, and an additional 88 studies were excluded because they were not relevant to the focus of our study. Fourteen studies were selected for further review, but seven were excluded as they did not specifically relate to robotic ectopic parathyroid glands and lacked sufficient details. As a result, seven studies remained for the final review. Figure 1 displays a PRISMA-style diagram illustrating the study selection process [11].

Table 1 summarises the outcomes and advantages of advanced surgical techniques for ectopic parathyroid gland resections, detailing results from 26 patients across various studies [12-18]. The data show that VATS offers reduced trauma and lower morbidity [13]. For instance, ectopic mediastinal parathyroid adenoma (EMPA) was successfully removed using robotic-assisted thoracic surgery (RATS) in conjunction with intraoperative PTH monitoring, which has been recommended for strict follow-up function monitoring [13]. After tumour removal, PTH levels fell to less than 50% of baseline, demonstrating the technique's effectiveness [13]. Among the patients, one individual had a small parathyroid adenoma on the left side, while another exhibited kidney stones, renal failure, and multiple brown tumours [14]. Remarkably, both patients were discharged on the third postoperative day without complications [14]. These outcomes underscore the safety and effectiveness of the robotic approach, which is associated with low postoperative pain and significant symptomatic improvement.

**Table 1:** Studies analysed in the reviews of robotic resection for an ectopic parathyroid gland. This table provides a summary of robotic resection outcomes for ectopic parathyroid glands. It summarises a number of studies, including patient demographics, surgical procedures, surgical technique advantages, preoperative conditions, operating times, blood loss, and postoperative results (0 = no complications, 1 = complications). PTH, Parathyroid hormone; iPTH, Intraoperative parathyroid hormone; RAP-EPRL, Robotic-assisted parathyroidectomy with en bloc right thyroid lobectomy; TAC, Transaxillary cervical; TTM, Transthoracic mediastinal parathyroidectomy (robotic surgical approaches used for parathyroidectomy)

Type of Paper	Author Name	Year	Country	Number of Patients	Age Range	Gender (M/F)	Type of Surgery	Number of Arms/Ports Used	Advantages	Before Surgery	Operation Time (Minutes)	Blood Loss	Outcome (0 = No Complication, 1 = Complication)
Case Report	Iijima et al. [12]	2022	Japan	1	53	F	da Vinci robotic	3-port robotic partial resection	Shorter hospital stays, better cosmetic outcomes	Mild hypercalcemia value and intact PTH level	76 (console time: 16)	Less	0
Retrospective Case Series	Van Dessel et al. [13]	2011	Belgium	2	34, 66	F, M	da Vinci robotic	3-arms	3D view, stable camera platform, fine dissection with wrist-free mobility	PTH high in both patients	65, 82	-	0
Case Report	Mansour et al. [14]	2019	USA (Ohio)	2	70	F	RATS	3-arms	-	-	183, 90	5-10 mL	0
Case Report	Ward et al. [15]	2017	USA (New York)	5	31-65	F (80%)	da Vinci robotic	3-4 arms	intraoperative parathyroid hormone decreasing ~50% from baseline after 10 minutes	Primary hyperparathyroidism from a lower mediastinal ectopic parathyroid	-	-	0
Case Series	Karagkounis et al. [16]	2014	USA	14	TAC: 47.3 ± 12.5; TTM: 48.2 ± 16	TAC: 7F, 1M; TTM: 4F, 2M	TAC and TTM	TAC-2 and TTM-2	Cosmetic benefits, precision, and minimally invasive techniques	Enlarged parathyroid glands	184 ± 58 minutes	-	TAC: 1; TTM: 1
Case Report	Katz et al. [17]	2012	USA	1	48	F	da Vinci robotic: RAP-EPRL	Arms 4, port 1	Reduced complications, patient satisfaction	Hyperparathyroidism, parathyroid adenoma	2-3 hours	<50 mL	0
Case Report	Ohara et al. [18]	2023	Japan	1	53	F	da Vinci Xi	8 mm ports	3D view, tremor filtering, offering full rotation, 7 degrees of freedom	Mediastinal tumour, nonfunctional pituitary adenoma, PTH high and calcium	76 (console time: 46)	Minimal	0

Additionally, advanced techniques, such as RATS and radionuclide-guided thoracoscopic surgery, show greater effectiveness in removing ectopic parathyroid tumours compared to standard VATS [15]. In the transaxillary cervical (TAC) group, one patient developed a postoperative seroma that was managed conservatively, while in the transthoracic mediastinal (TTM) group, one patient experienced pericardial and bilateral pleural effusions, which required further intervention, but they fully recovered [16]. A case report also highlights the use of robotic-assisted parathyroidectomy via a single axillary incision, resulting in reduced complications, high patient satisfaction, and a safe outcome for a patient with pHPT caused by a parathyroid adenoma [17]. Furthermore, radio-guided robotic parathyroidectomy provides an effective method for removing ectopic parathyroid tissue, using versatile articulated instruments that allow for precise, capsule-safe lesion removal [18]. Overall, the narrative underscores the importance of strict postoperative monitoring, which is essential to ensure the long-term efficacy of these surgical techniques [19]. The table also reports the successful resection of seven ectopic intrathoracic parathyroid glands using VATS [20]. Furthermore, radio-guided robotic parathyroidectomy provides an effective method for the removal of ectopic parathyroid tissue, utilising versatile articulated instruments that allow for precise, capsule-safe lesion removal [21]. Overall, the narrative underscores the importance of strict postoperative monitoring, which is essential to ensure the long-term efficacy of these surgical techniques [22].

**Figure 2:**
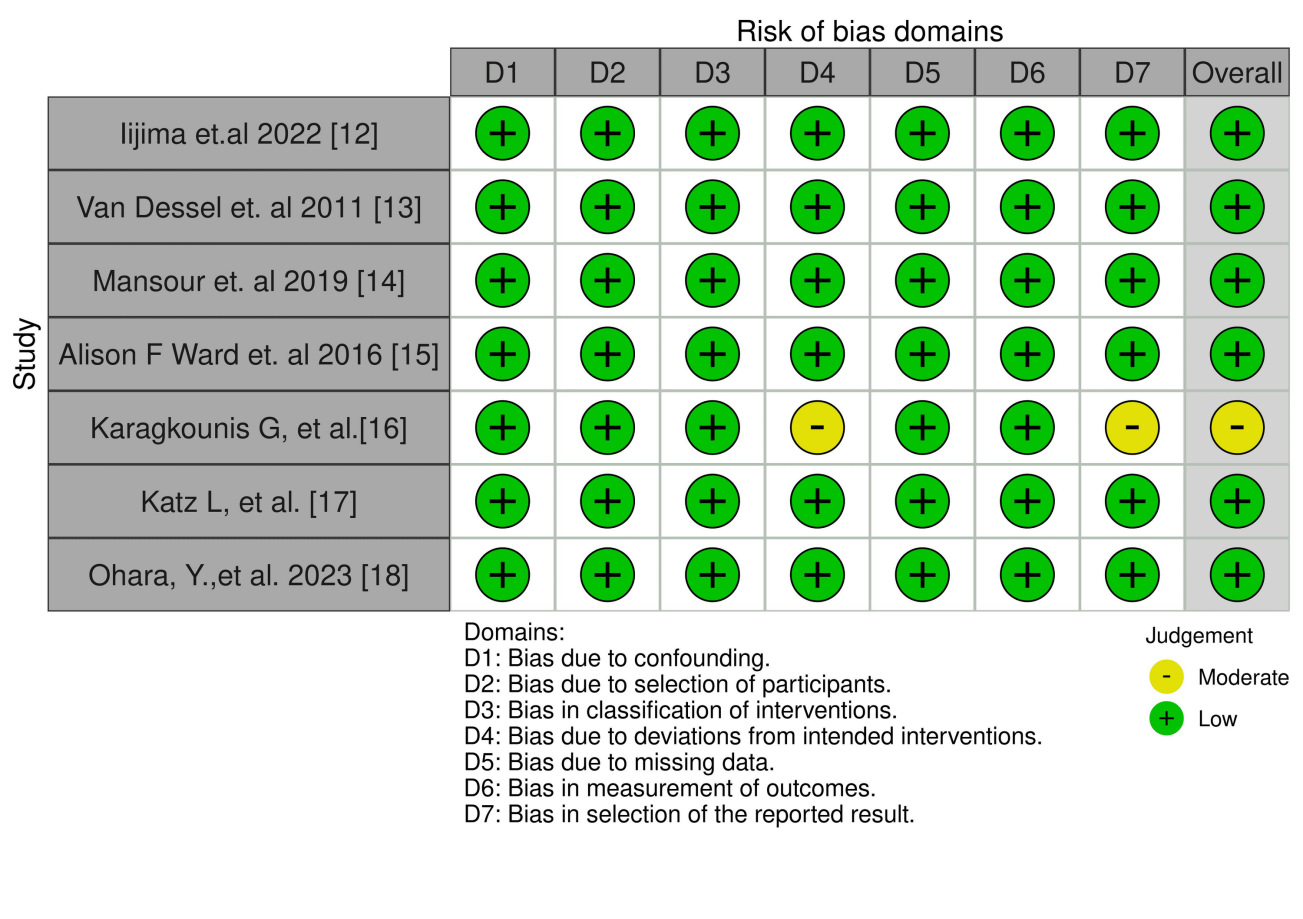
This graph shows the risk of bias for each study in our review using the ROBINS-I tool. Image credit: [23] ROBINS-I, Risk of bias in non-randomised studies of interventions

This is a risk assessment that summarises the author’s judgments on each risk of bias item for all studies included (Figure 2) [23]. Most studies were found to have a low risk of bias overall, as they addressed critical domains such as confounding, participant selection, and outcome measurement with transparency and rigour. Karagkounis et al. (2014) [16] stood out with a moderate risk, due to slight deviations in the intervention protocols and some concerns about the clarity of reporting. In summary, the majority of the studies demonstrated a high level of methodological quality, with minimal risk of bias across the domains assessed, confirming the reliability of their findings related to advanced surgical techniques for ectopic parathyroid gland resections.

Discussion

The studies frequently highlight the advantages of robotic systems, such as 3D imaging, a stable camera platform, precise dissection with flexible movement, and comfortable operating positions [24]. These factors contribute to reduced surgical trauma, decreased morbidity, shorter hospital stays, and improved cosmetic outcomes [24]. Robotic-assisted dissection is specifically beneficial for resection in remote and narrow anatomical locations, such as the mediastinum [24]. It offers a distinct advantage over non-robotic thoracoscopic procedures [24]. The presented studies focus on the effectiveness and advantages of different surgical methods for the resection of EMPAs. The use of robotic-assisted techniques, such as RATS and the da Vinci robotic system, is demonstrated in several cases.

Advantages and Disadvantages of Robotic Methods

Studies often highlight the benefits of using robotic systems in surgery [25]. These include features such as 3D imaging, a stable camera platform, precise dissection with flexible movement, and comfortable operating positions [25]. These factors contribute to reduced surgical trauma, decreased morbidity, shorter hospital stays, and improved cosmetic outcomes [25]. Robotic-assisted dissection is specifically beneficial for resection in remote and narrow anatomical locations, such as the mediastinum [25]. It has a clear advantage over non-robotic thoracoscopic procedures [25].

Robotic parathyroid surgery has some disadvantages. It takes longer than targeted open surgery because the surgeon needs more time to control the robot and lift a larger piece of skin. However, this extra time doesn't seem to cause any problems for patients [26]. Although there is a risk of brachial plexus injury associated with transaxillary robotic surgery, there have been no reported cases following transaxillary robotic parathyroidectomy [26]. However, rare instances have been documented in the literature for transaxillary robotic thyroidectomy [27-29]. Robotic parathyroidectomy is currently not cost-effective due to its high cost and limited evidence supporting its use. However, with growing competition in the robotic surgery market and impending patent expirations held by Intuitive Surgical Inc., costs are expected to decrease soon [30,31]. It is time-consuming and costly, and suitable only for carefully selected patients, with intensive training required for surgeons [32]. It's best suited for high-volume centres with experienced surgeons and may only be justified for select patients until costs decrease [33].

Histopathological and Intraoperative Monitoring Analysis

Histopathological findings reveal that small parathyroid gland tissues may remain in the surrounding thymus, highlighting the importance of comprehensive monitoring of parathyroid function during follow-up [33]. Intraoperative PTH monitoring is highlighted as a valuable tool to confirm a complete resection; this is important for ensuring the success of the surgery and preventing recurrence [33].

Patient Outcomes

In the studies, patients typically have positive results [34]. They undergo successful resections, quick recovery times, and minimal perioperative morbidity [34]. The decrease in postoperative pain scores and symptoms provides more evidence that these surgical methods are effective [34]. This innovative approach overcomes technical limitations and ensures no scarring on the neck [35].

Comparative Analysis

Examining different techniques, such as radio-guided robotic surgery, endoscopic and robotic parathyroidectomy, and VATS, provides insights into the advantages and disadvantages of each approach [36,37]. Research indicates that the choice of approach should consider factors such as scar concealment, postoperative cosmetic evaluation, and the extent of dissection [36,37]. Minimally invasive parathyroidectomy (MIP) is the recommended surgical procedure for treating pHPT [37]. It uses preoperative imaging to precisely locate the affected gland, reducing unnecessary surgical interventions [38]. Intraoperative methods ensure successful gland removal. MIP provides advantages such as faster recovery, shorter surgery times, and fewer complications [39].

Challenges and Considerations

Some researchers mention certain challenges, such as the prolonged elevation of calcium levels in patients with chronic renal insufficiency, or the need for strict follow-up to monitor parathyroid function [39]. One patient's unsuccessful cervical exploration and the need for further secondary investigations in others highlight the importance of accurate preoperative localisation and a clear diagnosis [40]. Robotic parathyroidectomy is safe and effective, with rare complications, like recurrent laryngeal nerve (RLN) palsy, hypoparathyroidism (<1%), bleeding, and wound infection [41,42]. According to this study, the transaxillary approach may lead to extended numbness and muscle stiffness, particularly affecting the regions surrounding the incision site, such as the pectoralis major and minor muscles, due to extensive dissection away from the gland. This finding aligns with previous literature, which highlights the association between increased dissection and the risk of nerve and muscle complications in these areas [42].

Limitations

Initially, there’s a possibility of overemphasising niche studies, potentially leading to the overlooking of the broader context within a field. Achieving a comprehensive understanding is challenging, and ensuring coverage of all relevant literature is difficult [42]. Depending solely on top journals might cause bias, disregarding valuable insights from alternative sources, such as conferences, potentially leading to biased research outcomes [42]. Furthermore, the selection of sources can be influenced by personal biases, which can compromise the review's objectivity.

## Conclusions

This review highlights the potential safety and efficacy of minimally invasive methods, particularly RATS and VATS, for the resection of EMPAs. While the reviewed studies demonstrate promising outcomes, including lower morbidity rates and improved cosmetic results, the findings are based on a small cohort of 26 participants from diverse centres using varied surgical techniques. This variability and limited sample size present significant challenges to generalising the results. Importantly, the review underscores the need for careful patient selection and tailored approaches, especially given the technical complexity of ectopic gland resection. However, the findings should be interpreted cautiously, as the limited number of cases may overemphasise the advantages of specific techniques while underrepresenting potential complications or long-term outcomes. To build on these insights, future research should focus on larger, multi-centre studies with standardised protocols and long-term follow-up. This will help validate the preliminary findings and clarify the sustainability and comparative advantages of these innovative surgical methods. Until such evidence is available, robotic-assisted and minimally invasive approaches should be considered promising but selectively applied in clinical practice.
